# Different sources of fat in starter and its effect on growth performance, blood parameters and immune system of calves

**DOI:** 10.1016/j.vas.2025.100451

**Published:** 2025-04-11

**Authors:** Hamid Paya, Mojtaba Hosseinzadeh, Akbar Taghizadeh, Ali Hosseinkhani, Karim Hasanpur, Maghsoud Besharati, Valiollah Palangi, Mehri Montazer Harzand, Maximilian Lackner

**Affiliations:** a Department of Animal Science, Faculty of Agriculture, University of Tabriz, Tabriz, Iran; b Department of Animal Science, Ahar Faculty of Agriculture and Natural Resources, University of Tabriz, Tabriz, Iran; c Visiting Researcher at Department of Life Sciences, Western Caspian University, Baku, Azerbaijan; d Department of Industrial Engineering, University of Applied Sciences Technikum Wien, Hoechstaedtplatz 6, 1200, Vienna, Austria

**Keywords:** Oilseeds, Saturated fatty acids, Unsaturated fatty acids, Suckling calf, Starter diet

## Abstract

Calf rearing from birth to weaning is a critical and sensitive period in dairy farming, as it determines the future of a herd. The aim of this work was to investigate the effects of different fat sources on growth performance, immune response and rumen and blood parameters in suckling calves. Forty female Holstein calves (average weight 40 kg, body score 3) were studied from birth to weaning (3 to 75 days). A completely randomized design with 4 treatments (10 replicates each) was used: i) control (no fat source), ii) coconut oil (2 % saturated fat), iii) flaxseed oil (2 % linoleic acid), and iv) safflower oil (2 % linoleic acid). Calf performance was evaluated weekly and feces were examined daily. Rumen parameters (volatile fatty acids and pH), blood parameters (glucose, protein, urea and cholesterol) and immune response (white and red blood cells and immunoglobulin G) were assessed at the end of the trial. According to the results, the highest feed intake after 10 weeks was recorded in the safflower oil treatment, which differed significantly from the control treatment (p < 0.05). The highest weight gain of calves fed diets containing unsaturated fats was observed in the 8th, 9th and 10th weeks, which was significantly higher than in the control and treatment containing coconut oil (p < 0.05). The height of experimental calves was affected by fats, except in the 1st and 2nd weeks. The experimental treatments were not significantly different in terms of fecal scores during the entire experimental period (p > 0.05). The effect of the experimental treatments was not significantly dependent on the pH of the calves' rumen fluid during the experimental period. The total volatile fatty acid content was significantly affected by the treatment (p < 0.01) at the end of the experimental period. Blood parameters (glucose, protein and urea), red blood cells and immunoglobulin G of the calves were significantly affected by the treatment (p < 0.05). The results of this study show that the use of different fat sources, especially those with unsaturated fatty acids containing linoleic and linolenic acid, in the starter diet of calves improved growth performance, immune response and rumen/blood parameters in female Holstein calves compared to the other groups.

## 1 Introduction

The inclusion of fat sources in calf starter diets has gained increasing attention as a potential strategy to optimize growth performance and health of dairy calves. Although dietary fats are not considered a primary nutrient requirement, they play a critical role, especially those rich in essential fatty acids (EFAs) such as linoleic acid. These EFAs promote cellular growth and development in dairy calves (Saini & Keum, 2018) and can be converted into functional fatty acids that are important for hormone production and physiological functions (Brozić et al., 2024; Saini et al., 2018; Uyarlar et al., 2024).

Recent studies have investigated the effects of different fat sources on the growth performance and health of dairy calves. Khalilvandi-Behroozyar et al. (2023) found that calves fed calcium salts of fish oil had higher average daily gains, better feed efficiency and lower inflammatory markers compared to calves fed calcium salts of soybean oil or a mixture of both. The authors attributed these benefits to the n-3 fatty acids contained in fish oil, which have anti-inflammatory properties and promote growth.

Feed digestibility may be limited in suckling calves due to an underdeveloped rumen, so increasing their diet's energy density with fat sources may be beneficial. The addition of fats, such as oilseeds, can help to better assess nutrient requirements for growth and immune responses in newborn calves (El-Hafeez et al., 2017; Wu et al., 2023). Linseed oil, safflower oil and coconut oil are examples of fat sources for both saturated and unsaturated FA. Linseed oil is rich in omega-3 and alpha-linolenic acid and is mainly used to increase the content of these fatty acids in meat and dairy products as well as to improve disease resistance and immunity. The maximum amount of oil that can be used in the diet of calves is usually limited to 6 to 7 % of the dry matter. Using more than this amount may cause issues such as reduced fiber digestion, impaired rumen function, decreased feed intake and energy supply (Yousefinejad et al., 2021)..

Safflower seed oil, rich in UFAs (∼93 %), contains 75–80 % linoleic acid and small amounts of linolenic acid, making it nutritionally valuable. The increasing use of linoleic acid reduces cholesterol and manages (Čeh et al., 2020). Feeding safflower seed oil has been shown to reduce cholesterol, regulate blood sugar, and boost the immune system in calves (Jaudszus et al., 2016; Ogori et al., 2020).

The influence of EFAs on calf health has been demonstrated in several studies. Hill et al. (2011) showed that the addition of a commercial supplement containing coconut oil, linseed oil and acetic acid to milk replacer for suckling calves improved feed conversion ratio, feed intake and average daily weight gain while reducing the stimulation of inflammatory factors. Garcia et al. (2015) reported that the addition of EFAs to the starter diet increased linoleic and linolenic fatty acids in calf liver and decreased saturated fatty acids, but this had less effect on growth, immune system function and dry matter consumption. Karcher et al. (2014) found that the addition of linseed oil improved FCR and growth rate while decreasing interleukin genes in calves, suggesting that omega-3 fatty acids can modulate immune response and support growth.

Despite these findings, there are few reports on the use of fatty acids to modify growth performance and immune response in suckling calves. We hypothesize that the inclusion of essential fatty acids in the starter diet for suckling calves will improve growth performance, immune response, and rumen and blood parameters compared to diets with lower fatty acid content. The aim of this study is therefore to investigate the effects of different fat sources, especially those rich in essential fatty acids, on growth performance, immune response and rumen and blood parameters of female Holstein calves.

## 2 Materials and methods

### 2.1 *Experimental design and treatments*

This study was conducted at Azar Nagin Agroindustry and Animal Husbandry Company (Khosrowshahr, East Azerbaijan Province, Iran) with forty female Holstein calves with an average initial body weight of 40 kg and a body condition score of 3 from birth to weaning (3 to 75 days old). The study was conducted during October, November, and December. The maximum, minimum, and average temperatures in October were 25 °C, −1 °C, and 11.6 °C, respectively; in November, 17 °C, −6 °C, and 5.2 °C; and in December, 11 °C, −8 °C, and 1.5 °C, respectively. It is worth noting that the dairy farm is located in a semi-cold region. The Animal Care and Use Committee of University of Tabriz approved all animal experiments according to the Iranian Council of Animal Care. Calves were randomly assigned to one of four dietary treatments in a completely randomized design with 10 replicates per treatment. All calves were assigned to treatments and the trial began on the same day. They were housed individually in pens measuring 2.5 × 1.5 m. The experimental treatments consisted of 1) control: a starter diet without a fat source; 2) coconut oil: a starter diet supplemented with 2 % coconut oil containing saturated fatty acids; 3) flaxseed oil: a starter diet supplemented with 2 % flaxseed oil containing linoleic acid; and 4) safflower seed oil: a starter diet supplemented with 2 % safflower oil containing linoleic acid. The ingredients and chemical composition of the starter feed and the compound feed are shown in Table 1, while the fatty acid composition of the oils used in this study is shown in Table 2. The metabolizable energy of the starter was 2.98, which increased to 3.01, 3.00, and 3.01 with the addition of coconut oil, flaxseed oil, and safflower seed oil, respectively.

**Table 1:** Ingredient and chemical composition of starter and mixed diets.

Ingredients (%)	
Corn grain	56
Soybean meal	37.5
Wheat bran	3.5
Sodium bicarbinate	0.4
Salt	0.5
Vitamins and minerals premix*	2
Calcium carbonate	0.5
Alfalfa hay⁎⁎	10

Chemical compositiond DM basis	
ME (Mcal/kg))	2.98
CP (%)	20.2
NDF (%)	17.6
ADF (%)	8.8

⁎ Vitamins and minerals permix composition: phosphorus 90 g, calcium 190 g, magnesium 95 g, zinc 5 g, sodium 55 g, iron 0.5 g, manganese 1.5 g, copper 0.3 g, selenium 3 mg, vitamin A 550,000 IU/kg, vitamin E 0.5 *g*/kg, vitamin D3 120,000 IU/kg.

⁎⁎ 10 percent of dry alfalfa chopped into pieces of 1–2 cm was added to the starter diet of the calves from the 20th day after birth.

**Table 2:** Fatty acids composition of oils (%).

Item	Coconut oil	Linseed oil	Safflower oil
C6	1.15	ND	ND
C8	7.61	0.08	0.05
C10	5.83	0.04	0.02
C12	51.28	0.03	0.02
C14	18.17	0.28	0.78
C16	6.97	6.11	8.09
C18	2.87	9.42	4.69
C18:1	3.49	21.45	16.79
C18:2	1.25	19.26	63.93
C18:3	ND	41.38	3.28
Others	1.38	1.95	2.35

### 2.2 *Measurement of feed intake*

Feed intake was measured for each calf 10 times (once per week) during the entire experimental period. Feed intake was calculated by the difference between the amount of provided feed and the remaining feed of the same day (based on the dry matter) for each calf (Hill et al., 2009).

### 2.3 *Measurement of calves' height and weight*

Calves' height and weight were measured weekly with a tape meter and a digital scale (Ganjkhanlou et al., 2014).

### 2.4 *Fecal scoring*

Fecal scoring was recorded every morning before cleaning the bed. Calf feces as a criterion for calf health were evaluated using the method proposed by Ghasemi et al. (2017). The fecal quality was scored with five points, namely normal fecal (1), smooth to loose fecal (2), loose to watery fecal (3), watery mucosal, and slightly bloody fecal (4), and watery, mucosal, and bloody fecal (5).

### 2.5 *Ruminal fluid sampling*

To evaluate rumen pH and volatile fatty acids (VFAs) on the last experimental day, the ruminal fluid was sampled using an esophageal tube and a vacuum pump 3 h after morning feeding. The pH of the sampled rumen fluid was measured with a digital pH meter. Then, 1 cm³ of 25 % metaphosphoric acid was added to every 4 cm³ of the rumen fluid and frozen at −20 °C until the reading of VFAs by a gas chromatographer (Chrompack cp 9002; Ottenstein & Bartley, 1971).

### 2.6 *Sampling and analysis of blood*

On the last day of the experiment, blood was sampled from the caudal vein of fasting calves using heparin-containing VENOJECT tubes. Blood plasma was separated by centrifugation (3000 rpm for 15 min). The extracted plasma was kept frozen at −20 °C. Blood parameters (cholesterol, glucose, total protein, urea) were determined by commercial kits (Pars Azmoon, Iran) and an autolyser (Hitachi Ltd, Japan). The blood serum IgG of calves was determined using an ELISA reader (Koma Biotech Company, Korea) by the ELISA method (kit No. 0231,016 K). Red and white blood cells (RBC & WBC) were determined with a Neubauer chamber (Hemocytometer, counting grid).

### 2.7 *Statistical analysis*

Repetitive data (feed intake, body weight, and daily weight gain) were analyzed by SAS statistical software using the mixed procedure. Non-repetitive traits were analyzed with the analysis of variance by SAS statistical software using the GLM procedure. The following is the statistical model used in this study: Yij = μ + Ti + Pj + β (Xi − X) + εij, where Yij is the observation rate, μ is the mean, Ti is the treatment effect, Pj is the measurement period effect, β(Xi – X) is the covariate factor effect, and εij is the error.

#### 2.7.1 Repeated measures analysis for body weight

For the body weight trait, the AIC statistic was used to compare models and select random and fixed variables for inclusion in the final model. To determine whether including the random effect of the animal in the model would improve the fit, the Mixed procedure was run once with and once without the random effect of the animal. The variance-covariance structure was considered as the software default in this case. Including the random effect of the animal did not reduce the AIC value (2159.6 compared to 2159.6), indicating that incorporating this random effect did not provide any advantage, and thus it was not included in the model.

To compare the type of variance-covariance structure and select the appropriate type, all structures were compared as follows (Table 3):

**Table 3:** Numerical values of AIC model.

Default	2159.6
CS	2161.6
AR(1)	2159.1
TOEP	2091.2
UN	2064.6
ANTE(1)	2010.5
ARH(1)	2001.6

CS: Compound symmetry, Toep: Toeplitz. AR(1): Autoregressive(1), UN: Unstructured, ANTE(1): Antedependence, ARH(1): Heterogeneous AR(1).

For the main analysis, the most suitable model, which utilized the ARH(1) variance-covariance structure, was used, and the least squares means for the main effects were reported.

#### 2.7.2 Repeated measures analysis for growth performance

For the height trait, the AIC statistic was used to compare models and select random and fixed variables for inclusion in the final model. To determine whether including the random effect of the animal in the model would improve the fit, the mixed procedure was run once with and once without the random effect of the animal. The variance-covariance structure was considered as the software default in this case. Including the random effect of the animal did not reduce the AIC value (2159.6 compared to 2159.6), indicating that incorporating this random effect did not provide any advantage, and thus it was not included in the model.

To compare the type of variance-covariance structure and select the appropriate type, all structures were compared as follows (Table 4):

**Table 4:** Numerical values of AIC model.

Default	1114.5
CS	1116.5
AR(1)	1115.4
TOEP	1105.8
UN	1157.5
ANTE(1)	1111.3
ARH(1)	1111.6

CS: Compound symmetry, Toep: Toeplitz. AR(1): Autoregressive(1), UN: Unstructured, ANTE(1): Antedependence, ARH(1): Heterogeneous AR(1).

For the main analysis, the most suitable model, which utilized the TOEP variance-covariance structure, was used, and the least squares means for the main effects were reported.

Non-repetitive traits were analyzed with the analysis of variance by SAS statistical software using the GLM procedure. The following is the statistical model used in this study: Yij = μ + Ti + Pj + β (Xi − X) + εij, where Yij is the observation rate, μ is the mean, Ti is the treatment effect, Pj is the measurement period effect, β(Xi – X) is the covariate factor effect, and εij is the error.

## 3 Results and discussion

### 3.1 *Feed intake, height, weight, and fecal scores*

The results of the effect of different experimental treatments on the feed intake of suckling calves are shown in Table 5. Accordingly, the feed intake was found not to be significantly affected by the treatment in periods 1–8, but the diets containing safflower oil and linseed oil were consumed more in most periods. The highest feed intake rate was measured in the safflower oil treatment in the 10th period, which was significantly different from the control treatment (P < 0.05).

**Table 5:** The effect of inclusion of SFAs and UFAs in the Holstein calves starter diet on feed intake (kg).

	Treatments					
Week	control	coconut oil	linseed oil	safflower oils	SEM	p-value
1	0.129	0.127	0.130	0.131	0.002	0.959
2	0.170	0.170	0.172	0.172	0.003	0.984
3	0.210	0.214	0.212	0.215	0.002	0.882
4	0.282	0.285	0.291	0.288	0.002	0.736
5	0.377	0.383	0.389	0.387	0.002	0.211
6	0.472	0.483	0.485	0.484	0.004	0.641
7	0.592	0.605	0.631	0.625	0.006	0.101
8	0.786	0.805	0.814	0.835	0.010	0.428
9	0.978^c^	1.047^bc^	1.107^ab^	1.139^a^	0.016	0.001
10	1.118^b^	1.167^ab^	1.197^a^	1.209^a^	0.011	0.015

Means within a column with different subscripts (a, b, c) differ (P < 0.05).

Based on the results, the calves' feed intake increased gradually during the experimental periods, which can be attributed to increased digestive tract volume, fermentation, and feed intake compared to the first days after birth. Higher feed intake was recorded for calves fed diets containing linseed and safflower oils than those treated with coconut oil and the control diet. This high feed intake results from the better fatty acid profile of linseed and safflower oils, leading to an increase in feed efficiency and the improved growth characteristics of calves. The use of unsaturated fats increased feed intake and animal milk production (Rajabi et al., 2017; Shakeri et al., 2017), which corresponds to the present results. In most periods, the diet containing coconut oil (saturated fat) resulted in a slight increase in feed intake compared to unsaturated fat. Contrary to our results, Hill et al. (2015) reported a decrease in feed intake by adding saturated fat to the diet of calves. Movahed Nasab et al. (2022) presented evidence that the addition of linseed oil did not affect feed intake, daily weight gain, feed efficiency, and skeletal growth.

**Fig. 1:**
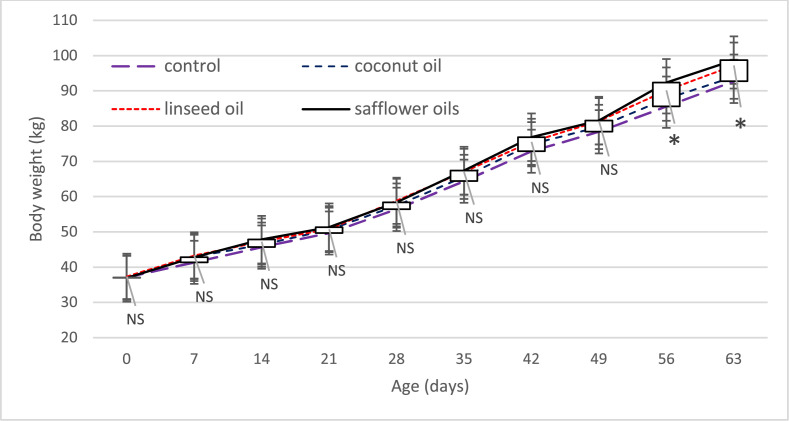
Impact of different fat sources on body weight of dairy calves. *P*-values symbols: NS is not significant,* < 0.05.

Effect of different fat sources on body weight in dairy calves fed different diets shown in Fig. 1. According to the results, the applied treatments did not significantly affect the weight gain of calves in the 1st to 7th periods, but they tended to be significant in the 7th period (P = 0.062). The weight of calves increased with increasing both the life period feed intake, and rumen development, but this weight gain was greater in calves fed diets containing UFAs. In the 8th, 9th, and 10th periods, the highest weight gain belonged to calves fed diets containing UFAs, which was significantly higher than the control and coconut oil treatments. This weight increase may be caused by supplying better energy and EFAs for calves and stimulating the development and growth of the rumen epithelial tissue by linoleic acid and linolenic functional fatty acids, leading to the improved average weekly weight of calves, which is similar to the results of Araujo et al. (2014). However, Ramezani et al. (2018) showed that the weight gain of calves was not influenced by the fat source used in the diets. The effect of inclusion of SFAs and UFAs in the Holstein calves starter diet on initial and weaning body weight and height reported in Table 6.

**Table 6:** The effect of inclusion of SFAs and UFAs in the Holstein calves starter diet on initial and weaning body weight and height.

	Treatments					
	control	coconut oil	linseed oil	safflower oils	SEM	p-value
Body weight, kg						
Initial body weight	40.1	40	40.2	40	0.173	0.975
Body weight at Weaning	95.7^b^	97.0^b^	100.8^a^	101.7^a^	0.630	0.001
Body height, cm						
Initial body height	76.69	76.68	76.65	76.63	0.131	0.239
Body height at Weaning	92.0^c^	93.4^b^	94.8^a^	95.0^a^	0.269	0.0007

Means within a column with different subscripts (a, b, c) differ (P < 0.05).

**Fig. 2:**
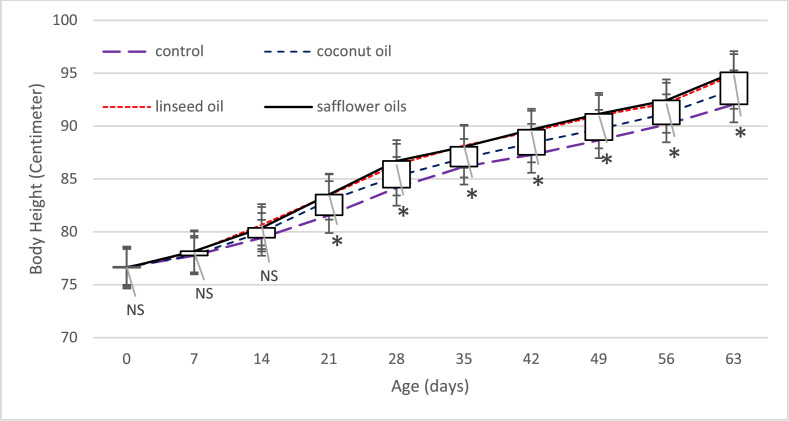
Efect of diferent fat sources on body height in dairy calves fed diferent diets. *P*-values symbols: NS is not significant,* < 0.05.

The impact of different fat sources on the body height in dairy calves fed different diets shown in Fig. 2. Based on the results obtained, the height of the experimental calves was affected by fats, except in the 1st and 2nd periods. In all periods, the highest height was measured in the calves fed diets containing linseed and safflower oils, which was significantly higher than the control and coconut oil treatments. According to the results of the calves' weight, the highest weight was obtained in the calves fed UFAs. Similar to these results, the increasing effect of dietary fats, especially UFAs, on calves' growth characteristics, such as height, was reported in previous studies (Ganjkhanlou et al., 2014; Hill et al., 2009).

Khalilvandi‑Behroozyar et al. (2023) reported that fat supplementation increased the intake of linoleic acid (the major n-6 FA), with the highest intake observed in calves fed the calcium‑salts of soybean oil compared to other treatments. Calves on the calcium‑salts of fish oil and mix of them consumed more long-chain n-3 FAs than those on other diets. Additionally, calves fed calcium‑salts of soybean oil and calcium‑salts of fish oil diets had higher starter feed and total dry matter intake compared to those mix and control diets throughout the experiment (days 3 to 65). Calves receiving the calcium‑salts of fish oil diet demonstrated the highest average daily gain, final body weight, and feed efficiency among all treatment groups.

Hill et al. (2007) demonstrated that specific fatty acid blends (C4:0, C8:0, C10:0, C12:0, C14:0, C18:2, and C18:3) partially replacing animal fats in milk replacers improved average daily gain (ADG), feed efficiency, and calf health. In their trials, calves fed starters containing sodium butyrate, canola oil, and coconut oil (FA1) or a proprietary fatty acid blend (FA2) showed faster and more efficient body weight gain compared to control calves. Consistently across all trials, ADG and feed efficiency were improved from 3 to 115 days of age by modifying the fatty acid profile of the starters.

The results (Table 7) did not show any significant differences in fecal scores between experimental treatments in the entire experimental period (p = 0.58). Calves usually suffer from diarrhea in the first 2–3 weeks of life and gain a higher fecal score. With the progress of life, however, the calves' fecal score decreased in all experimental treatments due to the immune system development, and the fecal score decreased gradually in this study. The inclusion of fatty acids in the diets of calves reduced the number of days with abnormal fecal profile (Fokkink et al., 2009).

**Table 7:** The effect of inclusion of SFAs and UFAs in the Holstein calves starter diet on fecal score.

	Treatments					
	control	coconut oil	linseed oil	safflower oils	SEM	p-value
Fecal score	3	2	1	1	0.33	0.58

### 3.2 *Rumen parameters*

This research indicated that nutritional treatments did not strongly impact the pH of calves' rumen fluid during the experimental period (p = 0.21) (Table 8). The highest and lowest pH values were obtained in calves fed the safflower oil diet and the control treatment. Rumen pH in suckling calves will not exceed 6 in the first 10 weeks after birth (Anderson et al., 1987), and this effect was observed in this study. The increase in pH results from the better efficiency of lactate-consuming bacteria, improvement of rumination resulting from more saliva secretion, and increased absorption of VFAs from the rumen environment. The higher rumen pH in calves fed diets containing UFAs probably results from the effect of these fats on the improvement of rumination.

**Table 8:** The effect of inclusion of SFAs and UFAs in the Holstein calves starter diet on rumen parameters.

	Treatments					
	control	coconut oil	linseed oil	safflower oils	SEM	p-value
pH	5.46	5.47	5.54	5.56	0.123	0.21
Total VFA (m mol)	86.32b	86.42b	88.7a	88.82a	4.44	0.0001
Acetate (m mol)	35.98b	36.05b	37.5a	37.76a	1.57	0.02
Butyrate (m mol)	16.63	17.76	19.11	20.18	2.14	0.22
Propionate (m mol)	29.58	29.57	29.12	28.82	1.56	0.64

Means within a column with different subscripts (a, b, c) differ (P < 0.05).

When the experiments were finished, the total VFAs were significantly affected by the treatment (p = 0.0001; Table 8). The highest and lowest contents of VFAs were obtained in diets containing safflower, flax, and coconut oils, as well as the control treatment (respectively), and no significant difference was observed in each of these treatments. The effect of treatments was significant on the acetate content (P = 0.02), but propionate and butyrate were not significantly affected by treatments. The ruminal VFAs are a good indicator in evaluating ruminal development and maturation. Ruminal metabolism is determined by the amount of produced VFAs and the methane content. Fat addition to ruminant diets reduces methane production and improves ruminal physiological conditions and even energy efficiency, in addition to increasing energy. Various types of fat supplements differently affect the results of rumen parameters depending on various factors such as the type and amount of fat supplements, fatty acid profile in fat supplements, rumen fluid properties, in vivo or in vitro experiments, and diet characteristics, which change the type and activity of rumen microorganisms (Yang et al., 2006). For this reason, there are different results about the effect of fats on rumen parameters. Le Roith et al. (2001) reported that rumen pH and total VFA concentration were not affected by fat supplementation, while Qiu et al. (2004) showed that soybean oil (UFA) increased acetate and total VFA concentrations and reduced propionate. Altogether, the results of this research indicate that the use of UFA-containing diets has beneficial effects on rumen parameters because of improved rumen microorganisms compared to SFAs.

### 3.3 *Blood parameters*

Table 9 shows the concentrations of glucose, protein, cholesterol, and urea parameters. According to the results, the calves' blood glucose were significantly affected by the treatment (p = 0.002). The calves fed a diet containing a fat source comprising higher glucose concentrations than those fed a fat-free source diet. As such, glucose concentrations were higher in calves on diets containing UFAs than in those being fed SFAs. According to Mashek et al. (2002), this result may be caused by the difference in the type of fatty acids affecting glucogenesis in ruminants' hepatocytes. The addition of fats, especially UFAs, to calves' diet could reportedly improve blood parameters and increase blood glucose compared to the control treatment (Dirandeh et al., 2016). Ghasemi et al. (2017) found that calves fed soybean oil had higher glucose levels, likely due to fat supplementation sparing glucose for other uses. In line with the findings of the present study, Khalilvandi‑Behroozyar et al. (2023) reported that calves receiving calcium‑salts of soybean oil and calcium‑salts of fish oil had higher glucose levels compared to the control group.

**Table 9:** The effect of inclusion of SFAs and UFAs in the Holstein calves starter diet on blood parameters.

	Treatments					
	control	coconut oil	linseed oil	safflower oils	SEM	p-value
Glucose (g/dl)	80.80^b^	84.00^ab^	94.80^a^	98.30^a^	10.30	0.002
Total protein (g/dl)	6.04^b^	6.66^a^	6.99^a^	7.06^a^	0.39	0.001
Cholesterol (mg/dl)	116.3	123.9	119.7	120.2	24.00	0.91
Urea (mg/dl)	13.6c	23.0a	20.1b	19.8b	2.06	0.0001

Means within a column with different subscripts (a, b) differ (P < 0.05).

The treatment significantly affected the protein content (p = 0.0001). The highest and lowest blood protein contents were obtained in calves fed with the safflower oil diet and control treatment, respectively. According to the results, no significant differences were found between fat-containing diets. Since the fat-containing diet was more consumed by calves, this increased diet consumption could elevate blood serum protein in calves fed a fat source. On the other hand, calves fed diets with UFAs contained higher protein levels because flax and safflower seeds are good sources of protein for animal nutrition. Moradi et al. (2018) reported that different sources of fat increased total blood serum protein, which corresponds to the outcomes of this research.

The results in Table 9 show the non-significant effect of treatments on blood cholesterol (p = 0.91). As indicated by the results, cholesterol concentration is higher in calves fed the fat source. Rajabi et al. (2017) reported that different fat sources increased blood cholesterol concentrations, which agrees with our results. On the other hand, cholesterol were higher in the calves fed the SFA-containing diet than those receiving diets containing UFA. This could be attributed to the role of the PPAR-α gene, which affects fat metabolism and cholesterol synthesis, absorption, and transportation in the body. PPAR-α gene activity increased in calves that received an UFA source and caused the absorption of cholesterols, which is in line with the results reported by Fernandez et al. (2005).

According to the results in Table 9, the effect of treatment was significant on blood urea concentrations in calves (p = 0.0001). The highest and the lowest blood urea were recorded in calves fed diets containing coconut oil and in the control treatment, respectively. High blood urea can be associated with high feed intake and probably more efficient rumen function (Khan et al., 2007). On the other hand, feeding with fats can increase blood urea as this result was obtained in this investigation. The blood urea concentration was significantly lower in calves fed UFA than in those that received SFA. This result might arise from the increased concentration of IGF-1 as a factor that increases protein synthesis and decreases protein oxidation in calves consuming linoleic acid. These effects improve both protein retention efficiency and body weight (Le Roith et al., 2001).

As shown in Table 10, the WBC count was not significantly influenced by treatment (p = 0.08). The highest and the lowest WBC counts were measured in the calves fed the safflower oil diet and in the control treatment, respectively. WBCs play an essential role in the body's immunity. Recent studies suggest that linolenic acid plays a more prominent role among essential fatty acids in the health and immunity of calves. Garcia et al. (2015) claimed that the consumption of EFAs increased linoleic acid and linolenic acid in the calf liver and improved the body's immune system function, whereas the use of SFA had less effect on calf immunity. As a precursor to cytokinins and eicosanoids with anti-inflammatory properties, linoleic acid is very suitable for livestock immunity. In a similar study (Ramezani et al., 2018), the use of UFAs improved the immunity of calves, which is similar to our results.

**Table 10:** The effect of inclusion of SFAs and UFAs in the Holstein calves starter diet on blood parameters.

	Treatments					
	control	coconut oil	linseed oil	safflower oils	SEM	p-value
White blood cells (1000/µl)	6.06	6.50	6.96	7.07	1.13	0.08
Red blood cells (1000/µl)	7.30^b^	8.03^b^	8.92^a^	8.88^a^	0.69	0.0001
Immunoglobulin G (mg/dl)	1977.7^b^	2370.7^a^	2619.2^a^	2560.8^a^	58.0	0.0001

Means within a column with different subscripts (a, b) differ (P < 0.05).

The treatment significantly affected RBC counts in calves (p = 0.0001; Table 10). The elevated RBC counts in calves fed fat-containing diets, especially UFAs, reflect the better immune response of these calves, leading to improved growth of calves. In fact, an active immune system increases resistance to diseases and supports nutrients for better performance and growth (Colditz, 2002). Ramezani et al. (2018) concluded that the use of conjugated linoleic acid increased RBC counts and improved the immunity of Holstein calves. However, our results showed that RBC counts in calves were significantly and positively affected by the source of UFAs, which is similar to the aforementioned study (Ramezani et al., 2018).

The results in Table 10 indicate the significant effect of treatments on the blood immunoglobulin G of the calves (p = 0.0001). The highest and lowest immunoglobulin G were obtained in calves fed linseed oil and control treatment, respectively. There were no significant differences between different fat sources. Feeding fats, particularly UFAs, increases blood IgG and strengthens the immune system in dairy calves, followed by the enhanced calf's resistance to many stresses in this sensitive period of life, thereby lowering deaths and more economical calf rearing. The results of experiments on the immune system functioning and the production of blood immunoglobulins using vegetable oils are generally contradictory. Previous studies reported reduced (Nudda et al., 2015), unaffected (Caroprese et al., 2009), (Mohammadi et al., 2015), and increased IgG concentrations in calves fed oil obtained from oil seeds. Momeni‐Pooya et al. (2022) presented evidence that starter supplementation with omega-3 fatty acids could be a strategy to improve the immune function of calves.

## 4 Conclusions

The results demonstrated that the highest feed intake, growth factors, the highest pH value, acetate, butyrate, and total fatty acids, the highest glucose and protein contents were observed in calves fed diets containing linseed and safflower oils. Altogether, it is concluded that the use of fat sources, especially those with UFAs containing linoleic and linolenic acids, in the starter diet of calves improves growth performance, immune response, blood parameters, and rumen parameters in female Holstein calves compared to the other groups.

## CRediT authorship contribution statement

**Hamid Paya:** Writing – original draft, Investigation. **Mojtaba Hosseinzadeh:** Investigation. **Akbar Taghizadeh:** Investigation. **Ali Hosseinkhani:** Methodology. **Karim Hasanpur:** Methodology. **Maghsoud Besharati:** Resources. **Valiollah Palangi:** Writing – review & editing. **Mehri Montazer Harzand:** Supervision. **Maximilian Lackner:** Writing – review & editing.

## Declaration of competing interest

The authors declare that they have no known competing financial interests or personal relationships that could have appeared to influence the work reported in this paper.
